# The impact of attrition on the representativeness of cohort studies of older people

**DOI:** 10.1186/1471-2288-10-71

**Published:** 2010-08-05

**Authors:** Samuel L Brilleman, Nancy A Pachana, Annette J Dobson

**Affiliations:** 1 University of Queensland, School of Population Health, Herston, QLD 4006, Australia; 2 University of Queensland, School of Psychology, St Lucia, QLD 4072, Australia

## Abstract

**Background:**

There are well-established risk factors, such as lower education, for attrition of study participants. Consequently, the representativeness of the cohort in a longitudinal study may deteriorate over time. Death is a common form of attrition in cohort studies of older people. The aim of this paper is to examine the effects of death and other forms of attrition on risk factor prevalence in the study cohort and the target population over time.

**Methods:**

Differential associations between a risk factor and death and non-death attrition are considered under various hypothetical conditions. Empirical data from the Australian Longitudinal Study on Women's Health (ALSWH) for participants born in 1921-26 are used to identify associations which occur in practice, and national cross-sectional data from Australian Censuses and National Health Surveys are used to illustrate the evolution of bias over approximately ten years.

**Results:**

The hypothetical situations illustrate how death and other attrition can theoretically affect changes in bias over time. Between 1996 and 2008, 28.4% of ALSWH participants died, 16.5% withdrew and 10.4% were lost to follow up. There were differential associations with various risk factors, for example, non-English speaking country of birth was associated with non-death attrition but not death whereas being underweight (body mass index < 18.5) was associated with death but not other forms of attrition. Compared to national data, underrepresentation of women with non-English speaking country of birth increased from 3.9% to 7.2% and over-representation of current and ex-smoking increased from 2.6% to 5.8%.

**Conclusions:**

Deaths occur in both the target population and study cohort, while other forms of attrition occur only in the study cohort. Therefore non-death attrition may cause greater bias than death in longitudinal studies. However although more than a quarter of the oldest participants in the ALSWH died in the 12 years following recruitment, differences from the national population changed only slightly.

## Background

Attrition due to the death and decline in the health of participants can cause particular problems in cohort studies of older people. Analyses of data from participants who continue in a study over many waves are potentially biased towards those who are healthy enough to do so. Many studies have identified associations between participant attrition and demographic or health related risk factors, and the authors have pointed out the need to distinguish between different types of attrition when examining these relationships [1-5]. The aim of this paper is to examine the effects of death and other forms of attrition on risk factor prevalence in the study cohort and the target population over time.

We look at two sources of attrition in older cohorts, referred to as death and non-death attrition (such as withdrawal and loss to follow up). First we consider several hypothetical situations in which a risk factor may be associated with differential mortality or differential non-death attrition. We then examine empirical data from the Australian Longitudinal Study on Women's Health (ALSWH) for women born in 1921-26, which show how these differential associations can occur in practice. Finally we use data from the five-yearly Australian censuses and National Health Surveys to illustrate the evolution of bias between the ALSWH and the target population over time.

## Methods

### Hypothetical situations

The hypothetical situations we consider are based on factors that might affect the generalisability of findings related to health service use by older participants in the ALSWH (the study cohort) to women of the same age group in the Australian population (the target population) after more than a decade. There were initial differences between the two groups, for example in level of education which could affect continuation in the study, health status, relative survival and use of health services.

To illustrate how biases might evolve we consider a hypothetical risk factor and examine changes in its prevalence over time in the target population and in the remaining cohort. We assume that the hypothetical risk factor is fixed at the individual level, for example country of birth. We also assume the prevalence of this risk factor is less than half. Bias is defined by the extent to which prevalence in the remaining cohort differs from prevalence in the target population. This will be affected by three factors: the extent of bias at the beginning of the study; any association between this risk factor and the risk of death, in the population or the cohort; any association between this risk factor and non-death attrition in the cohort.

The prevalence of the hypothetical risk factor in the *population *at time *t(m) *is defined as $p_{p,t(m)}=\frac{A}{(1-p_{p,t(m-1)})(1-I_{p,d})+A}$ where $A=p_{p,t(m-1)}\times \left[1-(RR_{p,d}\times I_{p,d})\right]$, *p_p, t(m-1) _*is prevalence of this risk factor in the population at time *t(m-1)*, *I_p, d _*is the incidence of death in the population for individuals without this risk factor between times *t(m-1) *and *t(m)*, and *RR_p, d _*is the relative risk of death in the population for individuals with this risk factor, compared to those without. The prevalence of the hypothetical risk factor in the remaining *cohort *at time *t(m) *is defined as $p_{c,t(m)}=\frac{B}{(1-p_{c,t(m-1)})(1-I_{c,d})(1-I_{c,a})+B}$ where $B=p_{c,t(m-1)}\times \left[1-(RR_{c,d}\times I_{c,d})\right]\left[1-(RR_{c,a}\times I_{c,a})\right]$, *p_c, t(m-1) _*is prevalence of this risk factor in the cohort at time *t(m-1), I_c, d _*and *I_c, a _*are the incidence of death and non-death attrition respectively between times *t(m-1) *and *t(m) *in the cohort for individuals without this risk factor and *RR_c, d _*and *RR_c, a _*respectively, are the relative risks of death and non-death attrition in the cohort for individuals with this risk factor, compared to those without. The length of time between *t(m-1) *and *t(m) *may be arbitrarily defined as any period for which the incidence rates apply, for example yearly.

For the hypothetical situations we assume the following numeric values. Prevalence of the hypothetical risk factor in the population at the beginning of the study is fixed at *p_p, t(0) _*= 0.25. When there is bias at the beginning of the study, prevalence of the hypothetical risk factor in the cohort is fixed at *p_c, t(0) _*= 0.20. All incidence rates (*I_p, d_*, *I_c, d _*and *I_c, a_*) are fixed at 0.10. If the hypothetical situation involves an association, this is modelled by setting the respective relative risk (*RR_p, d _*, *RR_c, d _*or *RR_c, a _*) equal to 2. In the case of no association, the relative risk is 1.

### Australian Longitudinal Study on Women's Health

The Australian Longitudinal Study on Women's Health (ALSWH) is a prospective nationwide study investigating factors relating to the health and well-being of Australian women. A random sample was drawn from the national Medicare health insurance database, which includes all Australian citizens and permanent residents. There was intentional over-sampling of women from rural and remote areas to provide reliable estimates for the less populated parts of the country. Further details of the study have been described elsewhere [6] and can be found at http://www.alswh.org.au. The study was approved by the ethics committees of the University of Newcastle and the University of Queensland and informed consent was received from all respondents and the data are openly available. Our analyses include women from the ALSWH cohort born in 1921-26. At the first survey in 1996, 12 432 women aged between 70 and 75 participated. We use data from Survey 1 (1996) through to Survey 5 (2008).

There are five categories of attrition at each survey (excluding Survey 1): respondent; dead; withdrawn due to frailty (frail); withdrawn due to other reasons (withdrawn); lost to follow up. The participant may formally withdraw herself from the ALSWH, or notification of withdrawal may be through a relative. The ALSWH seeks information on the reason for a withdrawal, and in this analysis we have included frailty as a special case. The reason for withdrawal cannot always be obtained. Information on the death of participants is obtained primarily through annual linkage to the Australian National Death Index. Additionally, the ALSWH is often notified by relatives of a deceased participant. Loss to follow up is used to describe all participants not otherwise categorised.

Country of birth was classified into three categories: Australia; other English speaking background; other. Highest educational qualification, which is considered here to be a proxy for socio-economic status, consisted of five categories: no formal qualification; any high school qualification; trade/apprenticeship; certificate/diploma; any university degree. Body mass index (BMI) was calculated as weight in kilograms divided by the square of height in metres and classified into four categories [7]: underweight (<18.5 kg/m^2^); acceptable (≥18.5, <25 kg/m^2^); overweight (≥25, <30 kg/m^2^); obese (≥30 kg/m^2^). Physical activity was classified into two categories: none or very low, corresponding to no activity or moderate activity once per week; low to very high, corresponding to moderate activity at least twice or vigorous activity at least once per week. Alcohol consumption was classified into four categories: non-drinker, rarely drinks; low risk drinker (1-2 drinks per day); risky or high risk drinker (3 or more drinks per day). Smoking status consisted of three categories: never smoker; ex-smoker; (current) smoker. Each woman's response to the statement "In general, would you say your health is:" was used as a measure of self-reported health with five categories: Excellent; Very Good; Good; Fair; Poor.

To model the association between risk factor levels at Survey 1 and attrition status at Survey 5 we used a multinomial logistic regression model, with 'respondent' as the reference category. For nominal scale risk factors the category with the highest frequency was chosen as the reference. For ordinal risk factors the end category associated with the best survival was chosen as the reference. All variables associated with attrition in univariate analyses were included in the multinomial logistic regression and only those with 95% confidence intervals not including unity for at least one category were retained in the final model.

### Population data

To illustrate these effects in practice we used two examples comparing ALSWH participants with national population data. Population data on country of birth for women born in 1921-26 were obtained from the 1996, 2001 and 2006 Australian censuses. In this age group there is little migration in or out of Australia. Country of birth was classified into two groups: non-English speaking countries and other, because of the relevance of language to use of health services and to correspond to the assumption in the hypothetical situations that the prevalence of the risk factor is less than a half.

Population estimates for the prevalence of smoking for women born in 1921-26 were obtained from the 1995 and 2005 Australian National Health Surveys. The National Health Survey is conducted through face-to-face interviews, and the sample design is such that within each State or Territory each individual has an equal chance of selection [8]. At the National Health Survey in 1995 and 2005 almost 900 women born in 1921-26 participated. Further details on the National Health Survey may be obtained from the Australian Bureau of Statistics at http://www.abs.gov.au.

For comparison to Australian population data, the ALSWH prevalence estimates require adjustment through the use of sample weights. These correct for the intentional over-sampling of women from rural and remote areas in the initial ALSWH sample.

## Results

### Hypothetical situations

**Figure 1:**
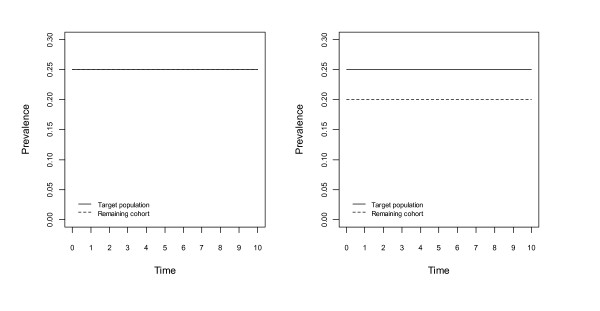
**Changes to prevalence of the hypothetical risk factor, assuming no association between the risk factor and the risk of death, in the population or the cohort; no association between the risk factor and non-death attrition in the cohort**. The left panel, Figure 1A, assumes no bias and the right panel, Figure 1B, assumes lower prevalence of the risk factor at the beginning of the study.

**Figure 2:**
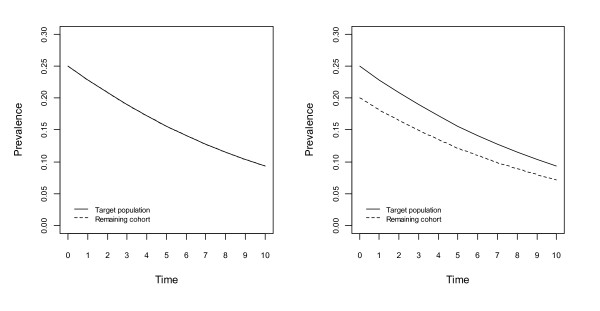
**Changes to prevalence of the hypothetical risk factor, assuming an association between the risk factor and the risk of death, in the population and the cohort; no association between the risk factor and non-death attrition in the cohort**. Figure 2A assumes no bias and Figure 2B assumes lower prevalence of the risk factor at the beginning of the study.

**Figure 3:**
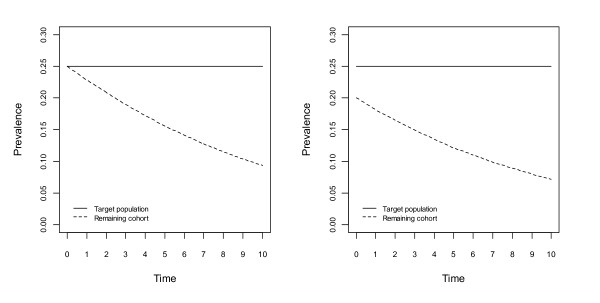
**Changes to prevalence of the hypothetical risk factor, assuming no association between the risk factor and the risk of death, in the population or the cohort; an association between the risk factor and non-death attrition in the cohort**. Figure 3A assumes no bias and Figure 3B assumes lower prevalence of the risk factor at the beginning of the study.

**Figure 4:**
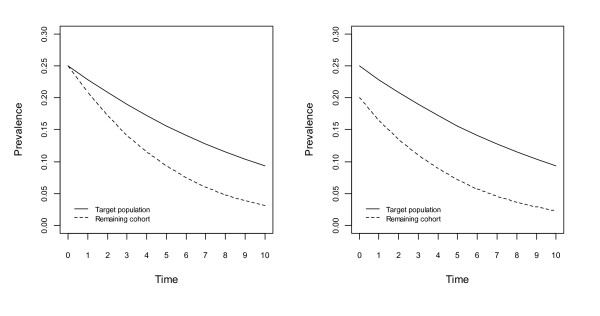
**Changes to prevalence of the hypothetical risk factor assuming an association between the risk factor and the risk of death, in the population and the cohort; an association between the risk factor and non-death attrition in the cohort**. Figure 4A assumes no bias and Figure 4B assumes lower prevalence of the risk factor at the beginning of the study.

Figures 1, 2, 3 and 4 show changes to the prevalence of the hypothetical risk factor, across a period of 10 time units, say 10 years. The left panel of each figure, labelled A, reflects no bias at the beginning of the study, whereas the right panel, labelled B, reflects the situation with initial bias.

In Figure 1 there is no association between the hypothetical risk factor and the risk of death in the population or cohort, nor is there an association between this risk factor and non-death attrition in the cohort. The prevalence of this risk factor in both the population and the cohort remains constant over time, and as a consequence the extent of the bias remains constant also.

In Figure 2 there is an association between the hypothetical risk factor and the risk of death in both the population and the cohort, but there is no association between this risk factor and non-death attrition in the cohort. For the population and the cohort, the strength of the association with the risk of death is assumed to be the same. If no bias exists at the beginning of the study as shown in Figure 2A, then this will remain the case over time. However if there is bias at the beginning of the study, Figure 2B, then this bias will evolve over time such that: if this risk factor is associated with an increased risk of death then the bias will decrease over time (prevalence in the population and the cohort will converge, as in Figure 2B); if this risk factor is associated with a decreased risk of death then the bias will increase over time (prevalence in the population and the cohort will diverge, not shown).

In Figure 3 there is no association between the hypothetical risk factor and the risk of death, however there is an association between this risk factor and non-death attrition in the cohort. Prevalence of this risk factor in the population remains constant over time. Prevalence of the risk factor in the cohort however, changes over time. In both panels of Figure 3 this risk factor is associated with higher non-death attrition and therefore prevalence in the cohort is decreasing. If the hypothetical risk factor was instead associated with lower non-death attrition then prevalence in the cohort would increase over time. These changes of prevalence in the cohort occur independently of the population prevalence.

In Figure 4, for the population there is a positive association between the hypothetical risk factor and the risk of death, and for the cohort there are positive associations between this risk factor and both the risk of death and non-death attrition. In both panels of Figure 4, greater change in prevalence of this risk factor occurs for the cohort than in the population, resulting in an increasing bias over time. The size of the bias is greater in Figure 3 and Figure 4 where there is a positive association between the hypothetical risk factor and non-death attrition; this is not the case in Figure 1 and Figure 2. If the associations are in opposite directions the bias may increase or decrease.

### Attrition in the Australian Longitudinal Study on Women's Health

Table 1 shows the attrition in the ALSWH at each survey. At Survey 5 approximately 45% of initial participants remain in the cohort, and of the women not in the cohort just over half have died (28.4% dead vs. 26.9% alive but no longer participating in the cohort).

**Table 1:** Attrition across Surveys 1 to 5 in the ALSWH, for women born in 1921-26.

Survey	Respondent	Dead	Frail	Withdrawn	Lost to Follow Up
1	100.0%	-	-	-	-

2	83.9%	4.5%	0.8%	4.7%	6.1%

3	69.6%	9.9%	2.6%	9.0%	9.0%

4	57.6%	18.4%	4.4%	10.9%	8.7%

5	44.7%	28.4%	5.1%	11.4%	10.4%

Total number of women at each survey is 12 432.

There were 8938 women with complete data on the Survey 1 risk factors that were significantly related to attrition status at Survey 5. They represent 71.9% of the initial sample. The missing data were mainly for BMI (11.0%), smoking status (7.4%) and country of birth (7.0%). Table 2 shows odds ratios (point estimates and 95% confidence intervals) for each of the risk factors estimated from a model that included all of them.

**Table 2:** Odds ratios from multinomial logistic model for different types of attrition, compared to response, at Survey 5 in the ALSWH for women born in 1921-26.

			Odds Ratio (95% Confidence Interval - bold indicates exclusion of 1)							
**Risk Factor**	**N**	**%**	**Dead** **(N = 2395)** **vs. Respondent**		**Frail** **(N = 410)** **vs. Respondent**		**Withdrawn (N = 942)** **vs. Respondent**		**Lost to Follow Up (N = 864)** **vs. Respondent**	

Country of Birth										

Australia	6893	77.1	1.00		1.00		1.00		1.00	

Other English speaking	1202	13.5	0.98	(0.84,1.15)	0.97	(0.70,1.33)	0.95	(0.76,1.19)	1.18	(0.95,1.47)

Other	843	9.4	0.95	(0.78,1.16)	1.17	(0.83,1.67)	**1.93**	**(1.55,2.42)**	**2.03**	**(1.62,2.54)**

Highest Qualification										

University	377	4.2	1.00		1.00		1.00		1.00	

Certificate/Diploma	728	8.1	1.24	(0.89,1.72)	1.05	(0.55,2.00)	1.54	(0.89,2.68)	0.81	(0.52,1.27)

Trade/Apprenticeship	327	3.7	1.12	(0.75,1.66)	1.49	(0.73,3.04)	**2.27**	**(1.25,4.13)**	1.10	(0.66,1.83)

Any high school	4665	52.2	1.31	(0.99,1.74)	1.19	(0.69,2.05)	**2.28**	**(1.41,3.70)**	1.17	(0.81,1.68)

No formal qualification	2841	31.8	**1.51**	**(1.13,2.01)**	1.41	(0.81,2.47)	**3.54**	**(2.18,5.76)**	1.20	(0.83,1.75)

BMI Classification										

Acceptable	4487	50.2	1.00		1.00		1.00		1.00	

Overweight	2959	33.1	**0.86**	**(0.76,0.97)**	0.91	(0.72,1.14)	1.07	(0.92,1.25)	1.11	(0.94,1.31)

Obese	1203	13.5	0.91	(0.77,1.07)	0.79	(0.57,1.10)	**0.62**	**(0.48,0.79)**	1.09	(0.87,1.37)

Underweight	289	3.2	**2.17**	**(1.62,2.92)**	1.72	(1.00,2.97)	1.04	(0.64,1.70)	0.77	(0.43,1.36)

Physical Activity										

Low to very high	6474	72.4	1.00		1.00		1.00		1.00	

None or very low	2464	27.6	**1.75**	**(1.55,1.97)**	1.08	(0.85,1.38)	**1.34**	**(1.14,1.59)**	1.19	(1.00,1.42)

Alcohol Consumption										

Low-risk drinker	3055	34.2	1.00		1.00		1.00		1.00	

Non-drinker	3007	33.6	**1.38**	**(1.20,1.58)**	**1.34**	**(1.02,1.75)**	1.15	(0.96,1.38)	1.13	(0.93,1.37)

Rarely drinks	2576	28.8	**1.21**	**(1.06,1.39)**	**1.43**	**(1.09,1.86)**	0.92	(0.76,1.10)	1.13	(0.94,1.37)

Risky or high-risk drinker	300	3.4	1.02	(0.76,1.37)	1.31	(0.74,2.32)	0.84	(0.54,1.31)	0.74	(0.46,1.18)

Smoking Status										

Never smoker	5624	62.9	1.00		1.00		1.00		1.00	

Ex-smoker	2673	29.9	**1.45**	**(1.29,1.64)**	1.10	(0.87,1.39)	1.00	(0.85,1.19)	**1.25**	**(1.06,1.48)**

Smoker	641	7.2	**2.73**	**(2.22,3.36)**	1.26	(0.80,1.97)	1.26	(0.92,1.72)	**1.82**	**(1.35,2.45)**

Self Reported Health										

Excellent	578	6.5	1.00		1.00		1.00		1.00	

Very good	2489	27.9	1.11	(0.86,1.42)	0.93	(0.56,1.55)	0.98	(0.73,1.33)	1.10	(0.79,1.53)

Good	3516	39.3	**1.57**	**(1.23,2.01)**	1.58	(0.97,2.55)	1.11	(0.83,1.49)	1.29	(0.93,1.78)

Fair	2014	22.5	**3.25**	**(2.52,4.19)**	**2.88**	**(1.75,4.74)**	**1.41**	**(1.03,1.93)**	**1.94**	**(1.37,2.73)**

Poor	341	3.8	**11.87**	**(7.92,17.79)**	**7.14**	**(3.54,14.41)**	1.73	(0.95,3.17)	**4.14**	**(2.38,7.20)**

In total 8938 women were included in the model, of which 4327 (48.4%) were respondents. Odds ratio estimates with a 95% confidence interval not including unity are shown in bold.

Compared to women who responded to Survey 5 those most likely to have died by Survey 5 had no formal educational qualification, were underweight, did little or no physical activity, rarely or never drank alcohol, were ex-smokers or current smokers, and reported having poorer health at Survey 1. Those who were overweight at Survey 1 were less likely to have died. Women most likely to withdraw due to frailty reported having poorer health at Survey 1, and drank alcohol rarely or never. Women most likely to withdraw due to reasons other than frailty were born in a non-English speaking country, had lower levels of education, did little or no physical activity and had poorer health; they were less likely to be obese. Women most likely to be lost to follow up were born in a non-English speaking country, were ex-smokers or current smokers, and reported having poorer health at baseline.

### Comparison with population data

**Figure 5:**
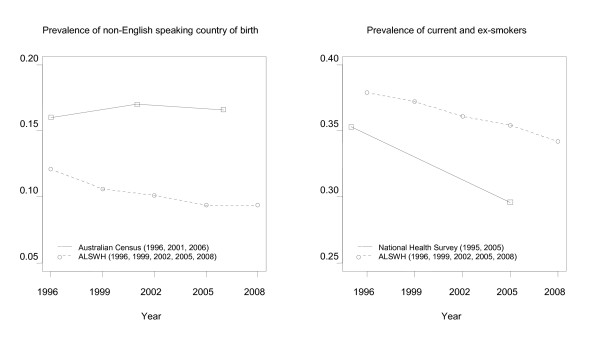
**Prevalence of a non-English speaking country of birth in the Australian Census and the ALSWH cohort (Figure 5A), and prevalence of current and ex-smokers in the Australian National Health Survey and the ALSWH cohort (Figure 5B)**.

Being born in a non-English speaking country is an example of a risk factor associated with non-death attrition but not the risk of death (i.e., similar to the situation depicted in Figure 3). Figure 5A shows the change in prevalence of a non-English speaking country of birth in the Australian population of women in this age group and the ALSWH cohort, over approximately the same ten years. Prevalence of a non-English speaking country of birth was lower in the ALSWH cohort than the national population in 1996 (0.121 vs. 0.160), 2001/2 (0.101 vs. 0.170) and 2005/6 (0.094 vs. 0.166). This corresponds to a bias of 0.039 in 1996, increasing to 0.069 in 2001/2, and 0.072 in 2005/6.

Smoking is an example of a risk factor associated with both the risk of death and non-death attrition (i.e., similar to the situation depicted in Figure 4). Figure 5B shows the change in prevalence of current or ex-smokers in the target population and the ALSWH cohort. Prevalence of current or ex-smokers was higher in the ALSWH cohort at both 1995/6 (0.379 vs. 0.353) and 2005 (0.354 vs. 0.296). This corresponds to a bias of 0.026 in 1995/6, increasing to 0.058 in 2005.

## Discussion

The Strengthening the Reporting of Observational Studies in Epidemiology (STROBE) Statement requires that the methods section in reports from studies should, if applicable, explain how loss to follow up was addressed [9]. At a minimum, mention should be made of the known disparity between the study cohort and the general population [10]. Often population estimates are unobtainable, and therefore a direct comparison between the study cohort and the target population may not be possible. A common approach to assess the potential for bias is to identify risk factors that are associated with attrition in the study cohort.

In this paper we have shown both theoretically and in practice, how bias may evolve as a consequence of both death and non-death attrition. We demonstrate that factors associated with the risk of death impact on bias differently from factors associated with other forms of attrition; the former impacting on both the study cohort and the target population, and the latter impacting on the study cohort alone. A review of data from ten longitudinal studies found health related bias due to attrition is particularly evident in adult and older adult cohorts, but not necessarily observable for data on younger people [10]. Particularly in studies of older people, where deterioration in health may cause deaths and a high level of non-death attrition, there are concerns that the remaining cohort may become increasingly unrepresentative of the target population.

From a theoretical perspective we need to consider the effect of the assumptions made in the hypothetical situations examined in this paper. First, prevalence of the hypothetical risk factor was assumed to be less than half. If prevalence is greater than half in both the population and the cohort, then the relationship between the direction of association with the risk of death and the direction in which the bias evolves is reversed. For example, if the hypothetical risk factor is associated with an increased risk of death in the population and the cohort, then in our hypothetical scenario the bias will decrease over time (as in Figure 2B). However if prevalence is greater than half in both the population and the cohort, then if this risk factor is associated with an increased risk of death the bias will in fact increase over time.

Second, the hypothetical risk factor was assumed to be fixed at the individual level. However, if this risk factor is not fixed then changes to the distribution may occur in both the target population and the cohort. Thus the potential for increasing bias is reduced as the target population may behave in a similar way as the cohort.

Attrition leads to missing data problems and as shown here and by others the missingness is not random, rather it is associated with the initial characteristics of the participants [1-5,11]. For non-death attrition, multiple imputation of missing data may be used provided that variables associated with the different types of attrition (including, if possible the type of attrition) are included in the imputation process. Random effects models and generalised estimating equations may be used for longitudinal analysis with data missing from participants who have dropped out [12]. However for attrition due to death it is necessary to consider separately the study participants who survive and those who die, for example by analysing their data separately or using a joint model for the longitudinal responses and the probability of survival [13]. More recent work suggests analysing the longitudinal data stratified by time of death [14]. For cohort studies of older people several of these methods may be needed to control bias due to attrition.

## Conclusions

Deaths occur in both the target population and the study cohort, while other forms of attrition occur only for the study cohort. Therefore non-death attrition is potentially a greater source of bias in longitudinal studies than death.

## Competing interests

The authors declare that they have no competing interests.

## Authors' contributions

All authors participated in the conception and design of the study. SLB constructed the mathematical models, did all the statistical analyses and wrote the first draft of the manuscript. NAP and AJD critically reviewed and revised it and AJD produced the final version. All authors read and approved the final version.

## Pre-publication history

The pre-publication history for this paper can be accessed here:

http://www.biomedcentral.com/1471-2288/10/71/prepub
